# Hepatitis C virus and risk of extrahepatic malignancies: a case-control study

**DOI:** 10.1038/s41598-019-55249-w

**Published:** 2019-12-19

**Authors:** Bo Liu, Yongxiang Zhang, Jun Li, Weihong Zhang

**Affiliations:** 1 Department of Infectious Diseases, The First Affiliated Hospital of Nanjing Medical University, Nanjing, China; 2 Department of Infection Management, The First Affiliated Hospital of Nanjing Medical University, Nanjing, China

**Keywords:** Cancer epidemiology, Hepatitis, Cancer

## Abstract

Epidemiological studies have demonstrated an increased risk of non-Hodgkin lymphoma (NHL) in patients with chronic hepatitis C virus (HCV) infection. Therefore, we investigated the risk of extrahepatic malignancies associated with HCV infection. Inpatients diagnosed with lymphoma, breast, thyroid, kidney, or pancreatic cancer (research group, n = 17,925) as well as inpatients with no malignancies (control group, n = 16,580) matched by gender and age were enrolled from The First Affiliated Hospital of Nanjing Medical University between January 2008 and December 2016. A case-control study was conducted by retrospective analysis. The difference in HCV prevalence was analyzed between the research group and the control group. Also, the research group was compared to the 2006 National Hepatitis C sero-survey in China. A total of 86 cases were positive for anti-HCV in the research group. Compared with the control group (103 cases were anti-HCV positive), no significant associations between extrahepatic malignancies and HCV infection were observed. Meanwhile, compared to the 2006 National Hepatitis C sero-survey, we observed a significant association between the chronic lymphoma leukemia/small lymphocytic lymphoma (CLL/SLL) and HCV seropositivity in females in the research group aged 1–59 years old (OR = 14.69; 95% CI, 1.94–111.01). HCV infection had a potential association with CLL/SLL in females aged 1–59 years old. Our study did not confirm an association between HCV infection and the risk of extrahepatic malignancies. In regions with a low HCV prevalence, the association between HCV infection and extrahepatic malignancies needs further investigation.

## Introduction

Hepatitis C virus (HCV) is a leading cause of liver-related mortality worldwide. Recent estimates have shown an increase in its seroprevalence over the last decade to 2.8%, with almost 185 million infections worldwide^1^. One-third of chronic HCV infections may progress to cirrhosis and hepatocellular carcinoma (HCC)^2^. Meanwhile, nearly 350,000 patients will die from HCV-related complications^3^. The HCV prevalence rate differs in different geographical regions, ranging from 1.20% in low-prevalence regions such as Latin America to 3.80% in high-prevalence regions such as the Middle East^4^. In China, the results of the Chinese National HCV serum prevalence survey in 2006 showed that the prevalence of anti-HCV was 0.43% in those aged 1–59 years old, which represents up to 10 million patients^5,6^.

Currently, cancer is among the top four causes of human mortality^7^. In a global epidemiological investigation, de Martel *et al*. found that 2 million cases (16%) of 12.7 million newly diagnosed cancer cases were attributed to exposure to infectious agents, with a higher rate recorded in developing countries^8^. HCC is the second leading cause of site-specific cancer-related death worldwide. In addition, HCV infection contributes to 80% of HCC cases^9^. Although HCV is a hepatotropic virus, several studies have shown that HCV may infect organs and tissues other than the liver, including peripheral blood cells, kidney, skin, oral mucosa, salivary glands, pancreatic tissues, heart, gallbladder, intestine, and adrenal gland tissues^10–15^. Moreover, HCV infection has been implicated in extrahepatic malignancies such as non-Hodgkin lymphoma (NHL), cholangiocarcinoma, pancreatic cancer, and oral carcinomas^16–19^. However, the association between HCV infection and extrahepatic malignancies among the Chinese population has not been investigated in depth. Thus, the aim of this study was to address this issue.

## Materials and Methods

### Study population and methods

**Figure 1:**
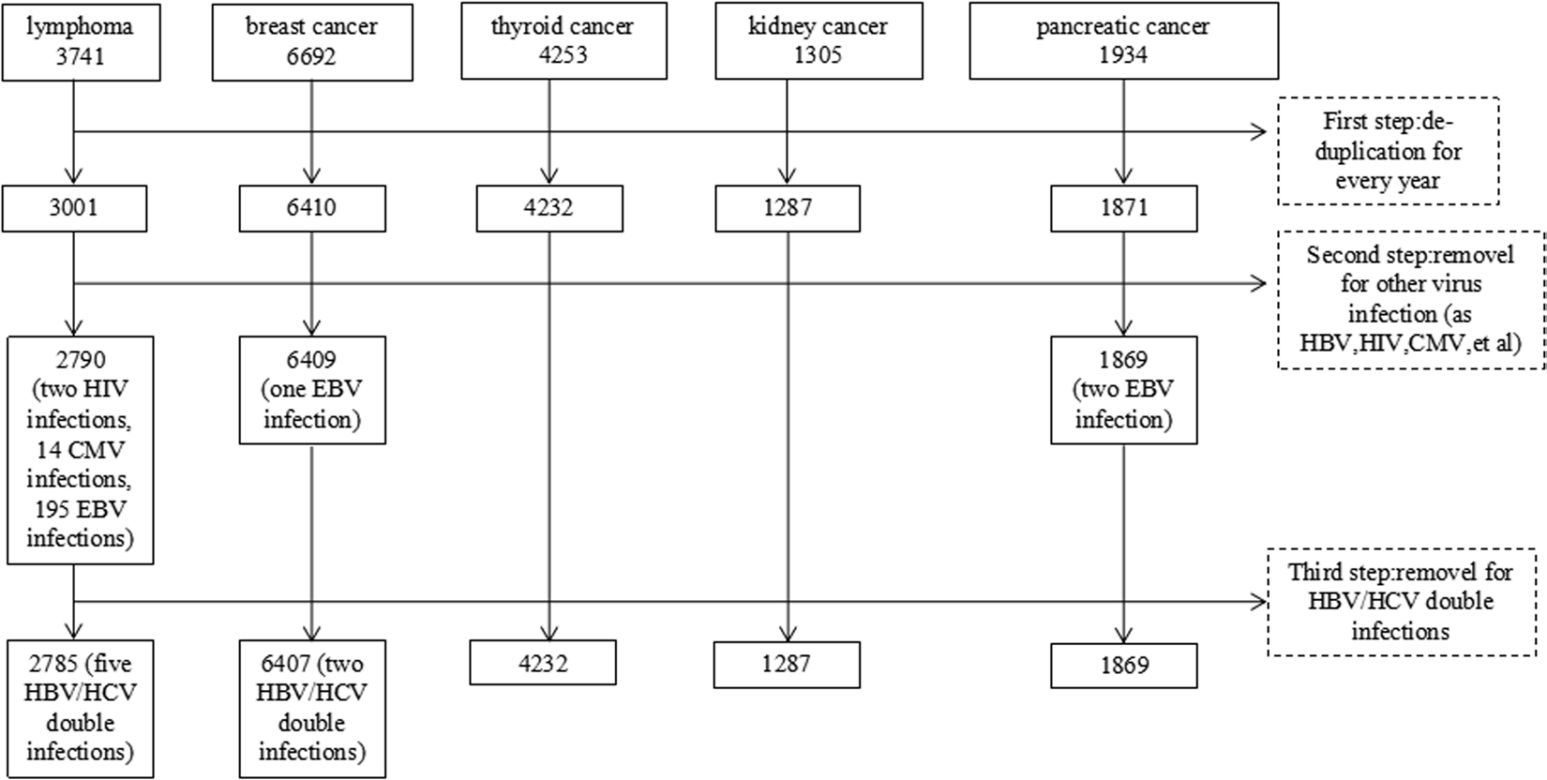
The screening process of five extrahepatic malignancies. The numbers of enrolled patients with lymphoma, breast, thyroid, kidney, or pancreatic cancer were 2785, 6407, 4232, 1287, and 1869, respectively (n = 16,580).

We conducted a case-control study by retrospective analysis. Inpatients diagnosed with lymphoma, breast, thyroid, kidney, or pancreatic cancer as well as cancer-free inpatients (control group) were enrolled at The First Affiliated Hospital of Nanjing Medical University from January 2008 to December 2016. The different malignancies were confirmed by pathological and histological examination. Accordingly, 17,925 patients were diagnosed with the above-mentioned extrahepatic malignancies. Next, all participants were screened for HCV infection as well as possible hepatitis B virus (HBV), human immunodeficiency virus (HIV), cytomegalovirus (CMV), and Epstein-Barr virus (EBV) infections. Participants with mixed infections (positive for anti-HCV antibodies as well as anti-HBV, anti-HIV, anti-CMV, and/or anti-EBV) were excluded (screening process seen Fig. 1).

Patients enrolled in the cancer group (research group) and the control group were matched for gender, age, and calendar year of selection by a ratio of 1:1. The different prevalence of anti-HCV seropositivity was analyzed between the research group (cancer patients) and the control group. Meanwhile, the data of the research group were compared to the data from the 2006 National Hepatitis C sero-survey in China. Lymphoma patients were classified for subtype-specific analysis using the World Health Organization Classification of Tumors of Hematopoietic and Lymphoid Tissues^20^. In this study, access to the patients’ data was approved by The First Affiliated Hospital of Nanjing Medical University Ethics Committee.An informed consent was obtained from all participants after an explanation of the protocol.

### Detection of viral index and definition of HCV infection

HBsAg, anti-HCV, and anti-HIV were detected by ELISA kits obtained from InTec Products, Inc., Xiamen, China. Patients with HCV seropositivity were defined as those with positive HCV antibodies. Anti-CMV and anti-EBV were detected by ELISA kits obtained from Beijing Baer Bioengineering Co., Ltd. and European Mongolian Medical Diagnostics (China) Co., Ltd., respectively.

### Statistical analyses

Statistical analyses were performed by IBM SPSS, version 22.0 (IBM, Armonk, NY). The mean and standard deviation were calculated for continuous variables with a normal distribution. The median (quartile) was calculated for continuous variables with a non-normal distribution. The chi-squared test was used to calculate the prevalence of anti-HCV seropositivity between the research group and the control group. The odds ratios (ORs) and corresponding 95% confidence intervals (95% CIs) were also measured to evaluate the association between HCV prevalence and the risk of extrahepatic malignancies. *P* values ≤ 0.05 were considered statistically significant.

## Results

### Characteristics of patients with extrahepatic malignancies

Among 16,580 patients with extrahepatic malignancies, there were 4593 males (27.73%) and 11,983 females (72.27%). The average patient age was 51 (SD = 15) years old. The 16,580 cancer-free participants (control group) had the same demographic characteristics in terms of age and gender due to the 1:1 matching ratio. The majority of patients with lymphoma, kidney cancer, or pancreatic cancer were males (59.39%, 64.10%, and 59.34%, respectively), and most of the patients with breast cancer or thyroid cancer were females (99.36% and 77.13%, respectively). The age ranges of patients with lymphoma, breast cancer, thyroid cancer, kidney cancer, or pancreatic cancer were 3–92 years old, 12–96 years old, 6–84 years old, 3–99 years old, and 6–92 years old, respectively. The mean age of patients with pancreatic cancer was the oldest, while the mean age of thyroid cancer patients was the youngest. In addition, the mean age of the lymphoma, breast cancer, and kidney cancer patients was around 50 years old (Table 1).

**Table 1:** Sex and age distributions of patients with extrahepatic malignancies.

Item	lymphoma	breast cancer	thyroid cancer	kidney cancer	pancreatic cancer
Sex (n, %)					
Male	1654 (59.39)	41 (0.64)	968 (22.87)	825 (64.10)	1109 (59.34)
Female	1131 (40.61)	6366 (99.36)	3264 (77.13)	462 (35.90)	760 (40.66)
Age (years)	53 ± 17	52 ± 12	43 ± 13	56 ± 14	62 ± 12

The majority of patients with lymphoma, kidney cancer, or pancreatic cancer were males, and most of the patients with breast cancer or thyroid cancer were females. The mean age of patients with pancreatic cancer was the oldest, while the mean age of thyroid cancer patients was the youngest.

During the nine consecutive years of this study, the number of cancer patients grew exponentially, especially for breast cancer and thyroid cancer patients. The number of patients with breast cancer increased from 359 in 2008 to 1272 in 2016, while the number of patients with thyroid cancer increased from 173 in 2008 to 1014 in 2016, as reported in Supplementary Fig. S1. The age of patients with extrahepatic malignancies had a normal distribution trend, as reported in supplementary Fig. S2. The peak age was 41–50 years old for patients with breast cancer or thyroid cancer (33.88% and 26.87%, respectively), while the peak age was 51–60 years old for patients with lymphoma or kidney cancer (24.74% and 25.95%, respectively). In contrast, the peak age of patients with pancreatic cancer was the oldest (61–70 years old, 34.40%).

### Comparisons of anti-HCV seropositivity between the research group and the control group

Among patients of both the research group and the control group, we observed that a total of 189 patients were positive for anti-HCV, 86 in the research group and 103 in the control group. Compared to the control group, the HCV prevalence was not higher in patients with extrahepatic malignancies (Table 2). In a subtype-specific analysis for lymphoma^20^, the HCV prevalence in patients with Hodgkin lymphoma (HL) (1.69%) was higher than that in patients with NHL (0.69%). The most common subtype of NHL was B-cell lymphoma. The most common types of B-cell NHL were diffuse large B-cell lymphoma (DLBCL; 60.80%), follicular lymphoma (FL; 12.24%), and mantle cell lymphoma (7.70%). The HCV prevalence among patients with chronic lymphoma leukemia (CLL)/small lymphocytic lymphoma (SLL) was the highest (1.44%) among the B-cell NHLs. Overall, HCV-seropositivity was not significantly associated with any subtype of lymphoma (Table 3). Furthermore, we conducted a subanalysis according to the sex distribution among patients aged 1–59 with extrahepatic malignancies. Compared to the national HCV sero-survey^5^, the prevalence of HCV was higher in female patients with CLL/SLL (5.56%), with a significant association between HCV-seropositivity and CLL/SLL (OR = 14.69, 95% CI: 1.94–111.01, *P = *0.001). On the other hand, we did not find significant associations between HCV-seropositivity and other subtypes of lymphoma (Table 4).

**Table 2:** Comparative results of anti-HCV for those with extrahepatic malignancies (research group) and the control group.

Extrahepatic malignancy	research group, n (%)	control group, n (%)	OR (95% CI)	*P* value
lymphoma	21 (0.75)	25 (0.90)	0.84 (0.47–1.50)	0.554
breast cancer	32 (0.50)	42 (0.65)	0.76 (0.48–1.21)	0.244
thyroid cancer	14 (0.33)	20 (0.47)	0.70 (0.35–1.39)	0.303
kidney cancer	5 (0.39)	7 (0.54)	0.71 (0.23–2.25)	0.774
pancreatic cancer	14 (0.75)	9 (0.48)	1.56 (0.67–3.61)	0.296

Among patients of both the research group and the control group, 86 and 103 were positive for anti-HCV, respectively. Compared to the control group, the HCV prevalence was not higher in patients with extrahepatic malignancies.

**Table 3:** Comparative analysis of the main histological subtypes of lymphoma between the lymphoma group and the lymphoma-related control group.

Category	Total (n)	anti-HCV (+), n (%)	OR (95% CI)	*P* value
Control group	2785	25 (0.90)	Reference	—
HL patients	178	3 (1.69)	0.53 (0.16–1.77)	0.292
NHL patients	2607	18 (0.69)	1.30 (0.71–2.39)	0.393
NHL				
precursor neoplasms	16	0 (0.00)	—	—
precursor lymphoid neoplasms	68	0 (0.00)	—	—
T/NK-cell lymphoma	431	3 (0.70)	1.29 (0.39–4.30)	0.675
B-cell lymphoma	2092	15 (0.72)	1.25 (0.66–2.39)	0.489
B-cell lymphoma				
DLBCL	1272	11 (0.86)	1.04 (0.51–2.12)	0.917
FL	256	2 (0.78)	1.15 (0.27–4.89)	0.849
MZL	143	0 (0.00)	—	—
CLL/SLL	139	2 (1.44)	0.62 (0.15–2.65)	0.515
mantle cell lymphoma	161	0 (0.00)	—	—
Burkitt lymphoma	63	0 (0.00)	—	—
other B-cell lymphoma	58	0 (0.00)	—	—

HCV-seropositivity was not significantly associated with any subtype of lymphoma. HL = Hodgkin lymphoma, NHL = non-Hodgkin lymphoma, DLBCL = diffuse large B-cell lymphoma, FL = follicular lymphoma, MZL = marginal zone lymphoma, CLL/SLL = chronic lymphoma leukemia/small lymphocytic lymphoma.

**Table 4:** Comparative analysis between those with the main histological subtypes of lymphoma aged 1–59 years old and the national survey according to sex distribution.

Category	male		OR (95% CI)	*P* value	female		OR (95% CI)	*P* value
	N	anti-HCV^+^, n (%)			N	anti-HCV^+^, n (%)		
national HCV data	37379	172 (0.46)	Reference		41367	165 (0.40)	Reference	
lymphoma	977	4 (0.41)	0.89 (0.33–2.40)	0.817	708	4 (0.56)	1.42 (0.53–3.84)	0.488
DLBCL	271	3 (1.11)	0.41 (0.13–1.30)	0.119	257	2 (0.78)	0.51 (0.13–2.07)	0.338
FL	70	0 (0.00)	—	—	64	0 (0.00)	—	—
MZL	26	0 (0.00)	—	—	26	0 (0.00)	—	—
CLL/SLL	18	0 (0.00)	—	—	18	1 (5.56)	14.69 (1.94–111.01)	0.001
marginal zone lymphoma								
MALT	10	0 (0.00)	—	—	19	0 (0.00)	—	—
SMZL	4	0 (0.00)	—	—	6	0 (0.00)	—	—
breast cancer	21	0 (0.00)	—	—	4766	26 (0.55)	1.37 (0.91–2.07)	0.135
thyroid cancer	820	2 (0.24)	0.53 (0.13–2.14)	0.363	2897	10 (0.35)	0.87 (0.46–1.64)	0.656
kidney cancer	466	1 (0.21)	0.47 (0.07–3.33)	0.435	275	2 (0.73)	1.83 (0.45–7.41)	0.390
pancreatic cancer	413	3 (0.73)	1.58 (0.50–4.98)	0.428	289	3 (1.04)	2.62 (0.83–8.25)	0.088

Compared to the national HCV sero-survey, the prevalence of HCV was higher in female patients with CLL/SLL, with a significant association between HCV-seropositivity and CLL/SLL (OR = 14.69, 95% CI: 1.94–111.01, *P* = 0.001). HL = Hodgkin lymphoma, NHL = non-Hodgkin lymphoma, DLBCL = diffuse large B-cell lymphoma, FL = follicular lymphoma, MZL = marginal zone lymphoma, CLL/SLL = chronic lymphoma leukemia/small lymphocytic lymphoma, MALT = mucosa-associated lymphoma tissue, SMZL = splenic marginal zone lymphoma.

## Discussion

The association between HCV infection and lymphoma, especially B-cell lymphoma of NHL, is the most studied subject in terms of HCV infection and extrahepatic malignancies^21–23^. In regions with a high HCV prevalence such as Southern Europe, including Italy and Spain, as well as Asian countries like Japan, HCV infection was obviously related to NHL^24,25^. However, in regions with a low HCV prevalence such as France and Canada, the association was not significant^26^. To date, in order to clarify the association between HCV infection and NHL, seven systematic reviews and/or meta-analyses have been published^22,23,27–31^. These analyses contained a total of 131 studies and five meta analyses, and they confirmed a significant association (OR range: 2–4). On the other hand, two analyses reported different results, especially when the subanalysis was performed according to region and race^23,29^. Meanwhile, HCV infection was only related to some subtypes of B-cell NHL such as DLBCL and marginal zone lymphoma^23^. Therefore, accumulating evidence has confirmed an association between HCV infection and NHL. However, there is no association for different regions, races, or subtypes of NHL.

In the current study, only 21 patients were positive for anti-HCV among 2785 patients with lymphoma. The prevalence of HCV was only 0.69% in NHL patients, which is even lower than that in patients with HL (1.69%). Although there were no significant differences for the prevalence of HCV between all five extrahepatic malignancies, including the main subtypes of lymphoma, and the national sero-HCV survey in men, the prevalence of HCV in CLL/SLL was significantly higher (5.56%) in females than in the corresponding control group from the national survey. Taken together, although this study did not confirm an association between HCV infection and most subtypes of NHL, female patients aged 1–59 years old with CLL/SLL had a higher prevalence of HCV infection (5.56%) than that from the national survey. Similarly, another recent Chinese study by Xiong *et al*. did not find a significant association between HCV infection and the main subtypes of lymphoma, except for splenic marginal zone lymphoma (SMZL), compared to the national sero-HCV survey^32^. It is important to note that although the subjects involved in the current study were matched for sex and age. However, we could not obtain detailed data from the national participants, especially for age-related information. This might have impacted the efficiency of our comparison; in particular, the peak of lymphoma showed an age-specific trend. In addition, there was only one CLL/SLL patient with positive anti-HCV, thus making it difficult to draw a solid conclusion.

Several studies have investigated the association between HCV infection and breast, thyroid, kidney, and pancreatic cancers in other countries^18,33–35^. Bruno *et al*. observed that some patients with HCC and anti-HCV positivity developed secondary cancers such as breast cancer^36^. However, later studies did not confirm such a significant correlation between HCV infection and breast cancer^33,37^. In addition, several research groups investigated the association between HCV infection and thyroid cancer, but the results are conflicting. Montella *et al*. and Antonelli *et al*. confirmed the presence of a significant assosiation, while Duberg *et al*. and Giordano *et al*. did not observe the same association^38–41^. In 2016, a meta-analysis by Wijarnpreecha *et al*.^35^ reported a significantly increased risk of kidney cancer among participants with HCV infection, with a RR of 1.86 (95% CI: 1.11–3.11). A major limitation in the meta-analysis by Wijarnpreecha *et al*. was the presence of significant heterogeneity between studies. Similarly, the association between HCV infection and pancreatic cancer is controversial as well^18,42,43^. With the exception to pancreatic cancer, limited information is available for the association between HCV infection and extrahepatic malignancies in China^44^. In this study, our results indicated that there was no significant association between HCV infection and breast, thyroid, kidney, as well as pancreatic cancers compared to the control group or the national sero-HCV survey.

The mechanism of HCV infection-induced NHL has been studied extensively. Upon treatment of HCV-infected lymphoma patients with interferon combined with ribavirin, lymphoma showed complete or partial regression^45,46^. A systematic review showed that 75% of those with lymphoproliferative disease and HCV infection could get complete regression after antiviral treatment^47^. Experimental research has explained HCV infection-induced NHL by three different mechanisms. De Re *et al*. have proposed that chronic antigen stimulation boosts B cell continuous reproduction^48^. Alternatively, the close attachment of envelope 2 protein of HCV to the CD81 receptor of B cells leads to polyclonal induction of immature CD27-B cells^49^. Finally, HCV infects B cells directly and is continuously replicated, resulting in gene mutation rearrangement, thereby promoting the abnormal clonal proliferation of B cells^50^.

In this study, we investigated the association between HCV infection and extrahepatic malignancies. However, our study had a few limitations that need to be addressed in future research. First, the research data were collected only from a single center; therefore, the results need to be confirmed by future multicenter studies. Second, this was a retrospective case-control study; thus, we could not provide a definite causal relationship between HCV infection and extrahepatic malignancies. Third, although age and sex were matched by a 1:1 ratio, other factors such as family history, environmental factors, and occupational factors were not considered.

In conclusion, compared to the control group, our case-control study did not confirm a significant association between HCV infection and the risk of lymphoma, including subtypes of lymphoma. However, HCV infection was significantly associated with CLL/SLL in females aged 1–59 years old, compared with controls from the national sero-HCV survey. On the other hand, our study did not find any significant association between HCV infection and the risk of other extrahepatic malignancies, including breast, kidney, and thyroid cancers. Therefore, in regions with a low HCV prevalence, the association between HCV infection and the risk of extrahepatic malignancies should be further studied in the future.

## Supplementary information

Supplementary Figure 1

Supplementary Figure 2

## Data Availability

The datasets generated during and/or analyzed during the current study are available from the corresponding author on reasonable request.
