# Supplementary material for: Hepatitis C virus and risk of extrahepatic malignancies: a case-control study
Source: Sci Rep. 2019 Dec 19;9:19444. doi: 10.1038/s41598-019-55249-w (PMC6923417; doi:10.1038/s41598-019-55249-w)

Supplementary Fig. 2. Age distributions of patients with extrahepatic malignancies.

Hepatitis C virus and risk of extrahepatic malignancies: a case-control study

Bo Liu,Yongxiang Zhang, Jun Li, Weihong Zhang

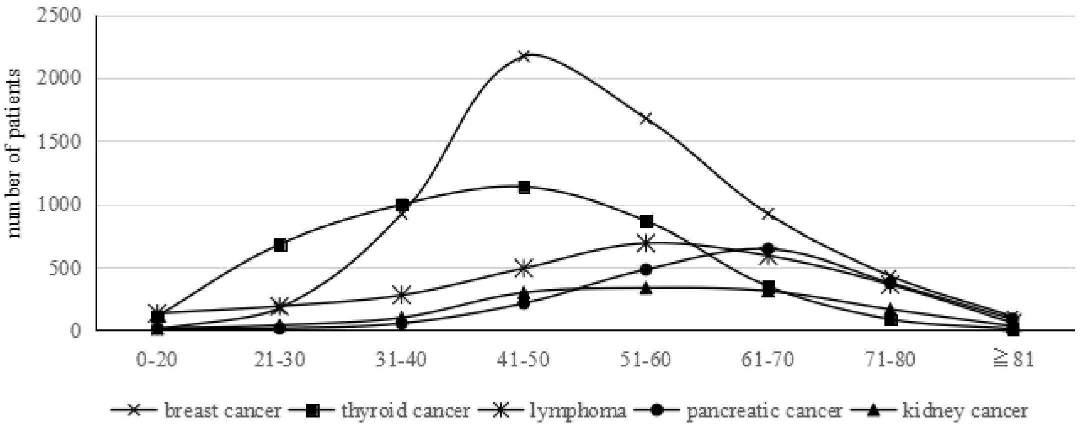

Supplement: Supplementary file 2 — Supplementary Figure 2 [file 41598_2019_55249_MOESM2_ESM.pdf]
